# Formation of Images on the Retina

**Published:** 1848-09

**Authors:** 


*Formation of Images on the Retina.*—Baly and Kirkes remark that it has been found by Volkmann that, in order to perceive the image of a bright object depicted on the retina of a human eye, it is not necessary to make an opening into the sclerotic and choroid coats, as formerly directed, for it can be perceived through these tunics almost as distinctly as through the transparent tissues of the eye of the white rabbit or other albino animal. For this purpose an individual should be selected in whom the eyes are large and prominent, and whose sclerotica possesses an unusual degree of transparency, as denoted by the bluish tint which it presents through the conjunctiva. When such an eye is directed as far outwards as possible, and a luminous object is then placed at the outside of it, at an angle from 80° to 85°, the image of this object may be detected at the inner angle of the eye appearing through the transparent sclerotica. Sometimes this image is so distinct that the inverted position in which the object is depicted on the retina may be clearly discerned.—*Med. Times.*

